# Supplementary figures and images for: Identification and Validation of PTGS2 Gene as an Oxidative Stress-Related Biomarker for Arteriovenous Fistula Failure
Source: Antioxidants (Basel). 2023 Dec 19;13(1):5. doi: 10.3390/antiox13010005 (PMC10812504; doi:10.3390/antiox13010005)

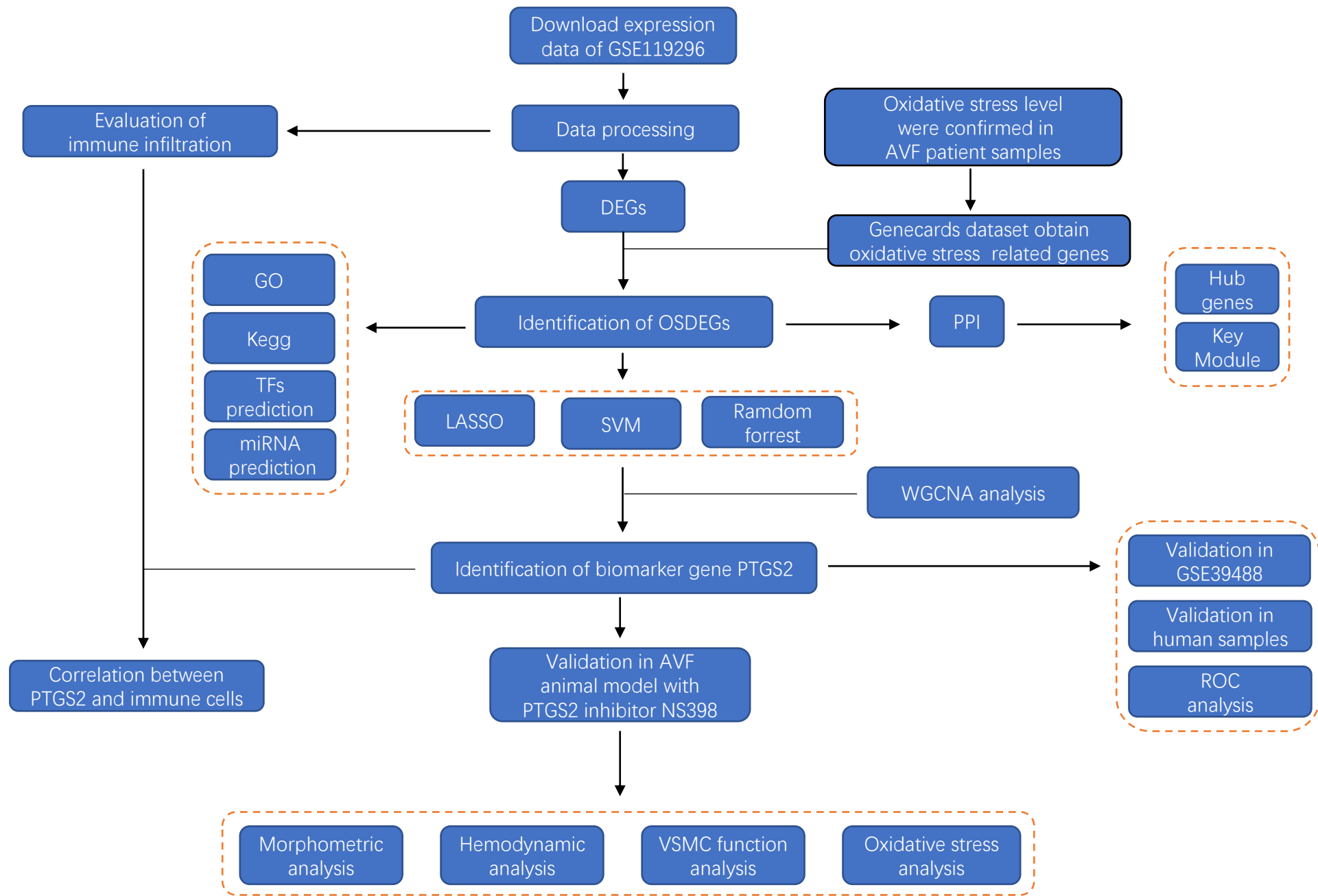

Supplement: Supplementary file 1 [file antioxidants-13-00005-s001.zip › supplementary-fig-1.pdf]

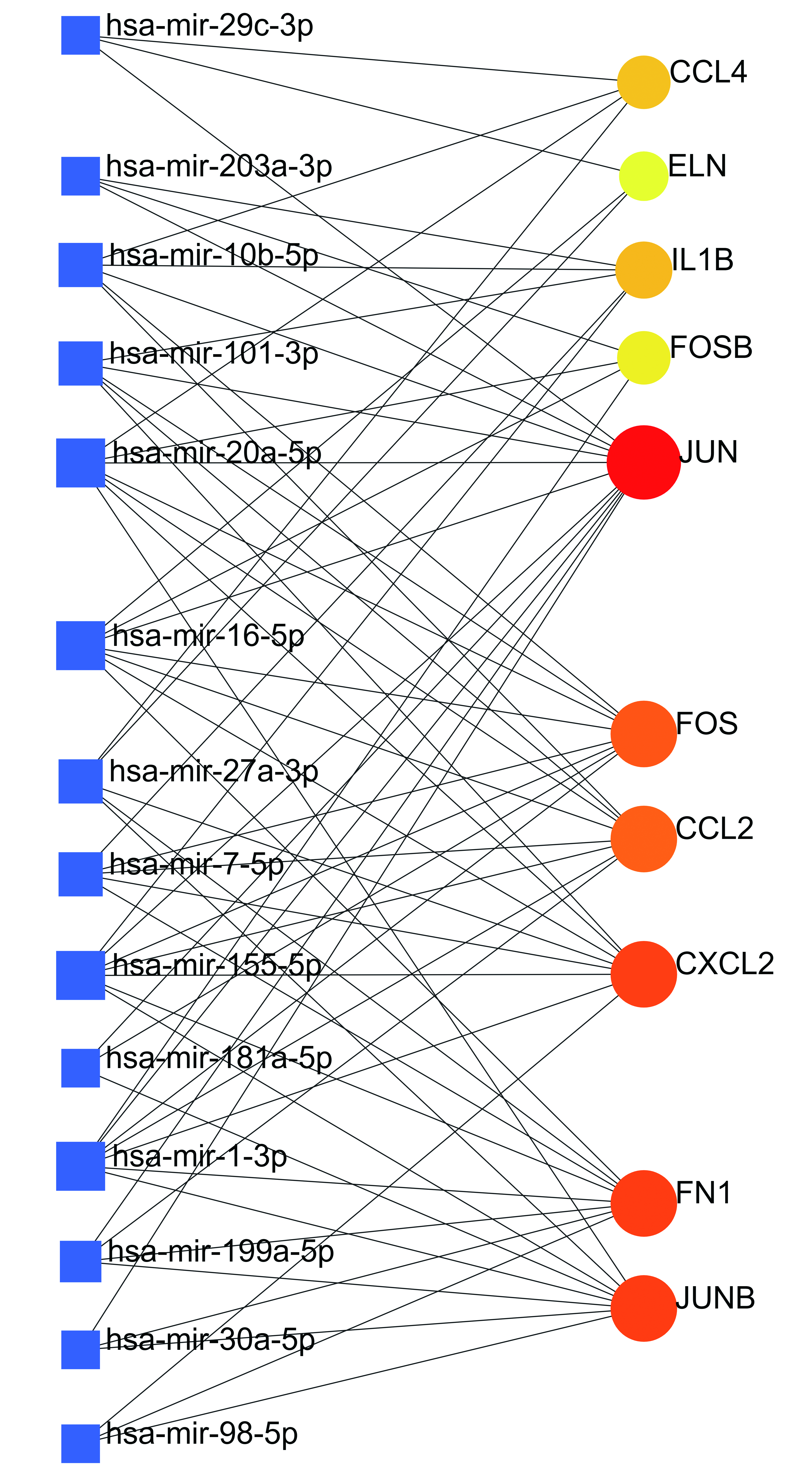

Supplement: Supplementary file 1 [file antioxidants-13-00005-s001.zip › supplementary-fig-3.tif]

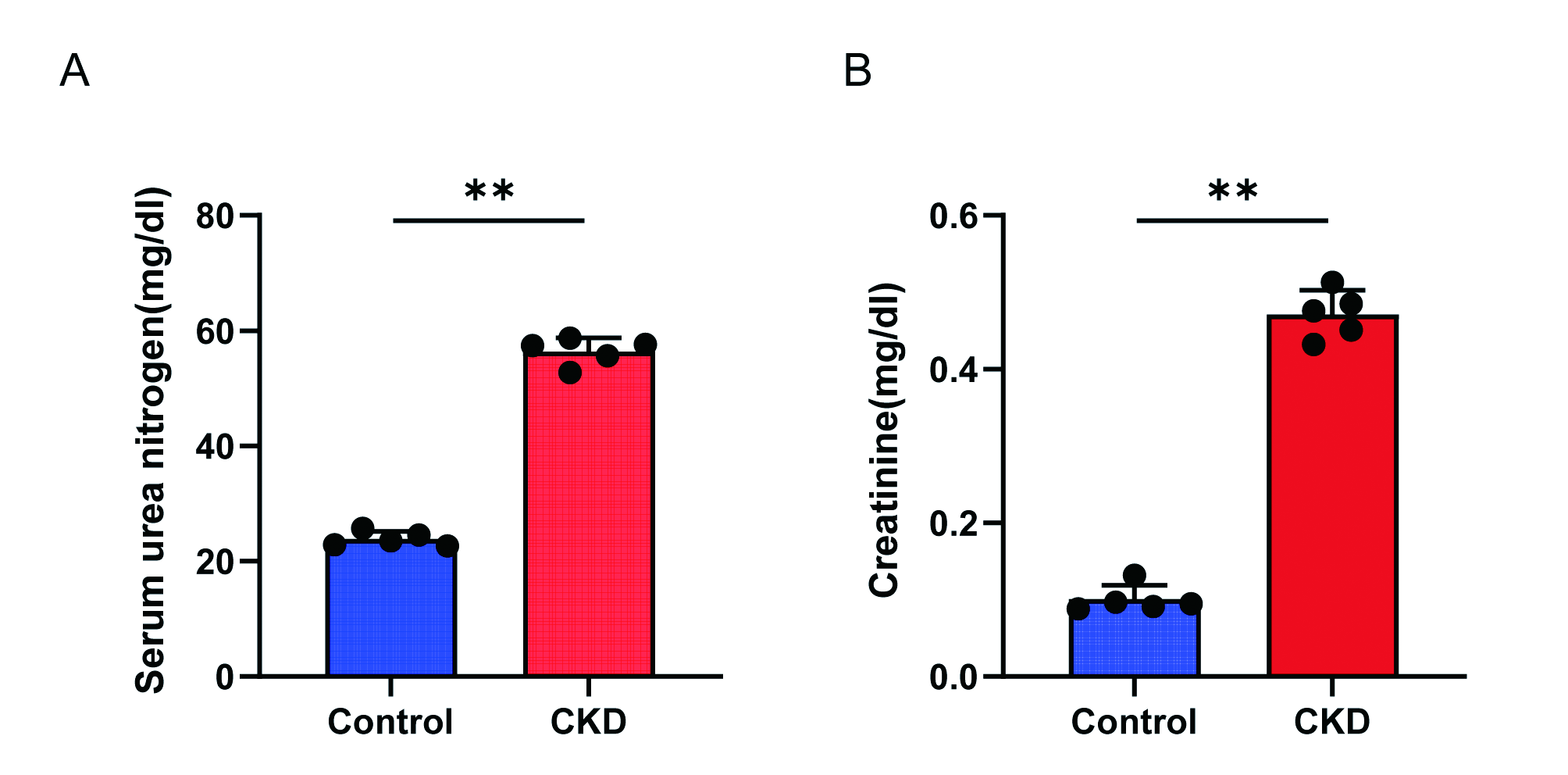

Supplement: Supplementary file 1 [file antioxidants-13-00005-s001.zip › supplementary-fig-4.tif]
